# Cytokeratin-18 is a sensitive biomarker of alanine transaminase increase in a placebo-controlled, randomized, crossover trial of therapeutic paracetamol dosing (PATH-BP biomarker substudy)

**DOI:** 10.1093/toxsci/kfae031

**Published:** 2024-03-23

**Authors:** Kathleen M Scullion, Iain M MacIntyre, Sian Sloan-Dennison, Benjamin Clark, Paul Fineran, Joanne Mair, David Creasey, Cicely Rathmell, Karen Faulds, Duncan Graham, David J Webb, James W Dear

**Affiliations:** Centre for Cardiovascular Science, University of Edinburgh, Queen’s Medical Research Institute, Edinburgh EH16 4TJ, UK; Centre for Cardiovascular Science, University of Edinburgh, Queen’s Medical Research Institute, Edinburgh EH16 4TJ, UK; Department of Pure and Applied Chemistry, Technology and Innovation Centre, University of Strathclyde, Glasgow G1 1RD, UK; Department of Pure and Applied Chemistry, Technology and Innovation Centre, University of Strathclyde, Glasgow G1 1RD, UK; Centre for Inflammation Research, University of Edinburgh, Institute for Regeneration and Repair, Edinburgh EH16 4UU, UK; Centre for Inflammation Research, University of Edinburgh, Institute for Regeneration and Repair, Edinburgh EH16 4UU, UK; Wasatch Photonics, Morrisville, North Carolina 27560, USA; Wasatch Photonics, Morrisville, North Carolina 27560, USA; Department of Pure and Applied Chemistry, Technology and Innovation Centre, University of Strathclyde, Glasgow G1 1RD, UK; Department of Pure and Applied Chemistry, Technology and Innovation Centre, University of Strathclyde, Glasgow G1 1RD, UK; Centre for Cardiovascular Science, University of Edinburgh, Queen’s Medical Research Institute, Edinburgh EH16 4TJ, UK; Centre for Cardiovascular Science, University of Edinburgh, Queen’s Medical Research Institute, Edinburgh EH16 4TJ, UK; Centre for Precision Cell Therapy for the Liver, Lothian Health Board, Queen’s Medical Research Institute, Edinburgh EH16 4TJ, UK

**Keywords:** keratin-18, microRNA, glutamate dehydrogenase, diagnosis, liver, DILI

## Abstract

Drug-induced liver injury (DILI) is a challenge in clinical medicine and drug development. Highly sensitive novel biomarkers have been identified for detecting DILI following a paracetamol overdose. The study objective was to evaluate biomarker performance in a 14-day trial of therapeutic dose paracetamol. The PATH-BP trial was a double-blind, placebo-controlled, crossover study. Individuals (*n* = 110) were randomized to receive 1 g paracetamol 4× daily or matched placebo for 2 weeks followed by a 2-week washout before crossing over to the alternate treatment. Blood was collected on days 0 (baseline), 4, 7, and 14 in both arms. Alanine transaminase (ALT) activity was measured in all patients. MicroRNA-122 (miR-122), cytokeratin-18 (K18), and glutamate dehydrogenase (GLDH) were measured in patients who had an elevated ALT on paracetamol treatment (≥50% from baseline). ALT increased in 49 individuals (45%). All 3 biomarkers were increased at the time of peak ALT (K18 paracetamol arm: 18.9 ± 9.7 ng/ml, placebo arm: 11.1 ± 5.4 ng/ml, ROC-AUC = 0.80, 95% CI 0.71–0.89; miR-122: 15.1 ± 12.9fM V 4.9 ± 4.7fM, ROC-AUC = 0.83, 0.75–0.91; and GLDH: 24.6 ± 31.1U/l V 12.0 ± 11.8U/l, ROC-AUC = 0.66, 0.49–0.83). All biomarkers were correlated with ALT (K18 *r* = 0.68, miR-122 *r* = 0.67, GLDH *r* = 0.60). To assess sensitivity, biomarker performance was analyzed on the visit preceding peak ALT (mean 3 days earlier). K18 identified the subsequent ALT increase (K18 ROC-AUC = 0.70, 0.59–0.80; miR-122 ROC-AUC = 0.60, 0.49–0.72, ALT ROC-AUC = 0.59, 0.48–0.70; GLDH ROC-AUC = 0.70, 0.50–0.90). Variability was lowest for ALT and K18. In conclusion, K18 was more sensitive than ALT, miR-122, or GLDH and has potential significant utility in the early identification of DILI in trials and clinical practice.

Drug-induced liver injury (DILI) is a major challenge for public health and drug development. DILI is a leading cause of acute liver failure (ALF) (case fatality of around 30%–35%), with about 30% of patients with ALF needing to receive a liver transplant with its associated risks and financial costs (Stravitz and Lee, 2019). In drug development, DILI is one of the leading causes of drug attrition and withdrawal of drugs during the development phase (Kullak-Ublick *et al.*, 2017). This can result in huge financial losses for the drug developer and reduces the availability of new medicines for patients with unmet clinical needs.

Medicines with a liability for producing DILI can be used safely and effectively. For example, immune checkpoint inhibitors (ICIs) have transformed the treatment of a range of cancers despite being well-established to produce DILI, especially when 2 ICIs are combined (Regev *et al.*, 2020). To safely use such medicines there may be a need for risk mitigation strategies to improve the risk/benefit balance. For example, if DILI could be identified earlier then targeted safety monitoring could be intensified, the causal medicine could be withheld, or its dose modified before harm occurs to the patient. Early detection of DILI is especially important when one or more medicines must be re-introduced after an episode of DILI, such as occurs when the treatment of tuberculosis (TB) requires re-introduction of effective but potentially hepatotoxic antimicrobials (Moosa *et al.*, 2022). Monitoring for DILI typically involves blood being taken in a healthcare facility with analysis in central hospital laboratories. Future strategies may decentralize monitoring with point of care analysis near to the patient, such as home monitoring for DILI in early phase clinical trials of selected medicines with a DILI liability or community monitoring for TB medicine DILI in low- and middle-income countries (LMICs).

To improve the diagnosis of DILI, there has been a global effort to identify and develop new biomarkers with enhanced sensitivity and/or specificity or that provide new mechanistic insights. Paracetamol (acetaminophen) is the most common cause of DILI, and is the classic example of *intrinsic* DILI, as the risk of injury is directly related to the dose due to hepatocellular injury being mediated by a toxic metabolite (Ramachandran and Jaeschke, 2017). Patients attending hospital following an acute paracetamol overdose have provided a well-characterized population to establish proof-of-concept that novel biomarkers such as cytokeratin-18 (K18) and microRNA-122 (miR-122) may be more sensitive than the standard biomarker alanine transaminase (ALT) (Dear *et al.*, 2018). Transatlantic collaborations such as Drug-Induced Liver Injury Network (DILIN), Predictive Safety Testing Consortium (PSTC), Safer and Faster Evidence-based Translation (SAFE-T), and Translational Safety Biomarker Pipeline (TransBioLine) have demonstrated the potential utility of multiple protein and RNA circulating biomarkers (including K18 and miR-122) in patients with *idiosyncratic* DILI (DILI resulting from characteristics of the individual affected and not clearly related to drug dose or duration [Fontana, 2014]) secondary to therapeutic use of a range of medicines (Church *et al.*, 2019). *Idiosyncratic* DILI is a rare event and these studies are, therefore, case-control by necessity, with the cases having well-established liver injury. It remains unclear whether new biomarkers can identify *idiosyncratic* DILI before standard biomarkers are increased. In this study, we used paracetamol as a model of chronic dosing to provide proof of concept as to whether new biomarkers could potentially identify DILI before standard markers are increased.

Reported in a 2021 paper, we identified a 3-biomarker panel (K18, miR-122, and glutamate dehydrogenase [GLDH]) which accurately detected DILI after acute paracetamol overdose (Llewellyn *et al.*, 2021). Previous studies have also investigated the sensitivity of these biomarkers in tuberculosis treatment-related DILI in European and Ugandan populations (Rupprechter *et al.*, 2021). Here, we report the results of testing this biomarker panel in a predefined analysis of a double-blind, placebo-controlled, crossover trial of paracetamol at therapeutic doses, which acts as a model for early phase drug trials (MacIntyre *et al.*, 2022). This allowed testing of whether one or more of the new biomarkers can detect DILI earlier than ALT in a multiple dosing trial and, therefore, may be valuable in future trials and clinical practice.

## Materials and methods

###  

####  

##### Study design

This study was a substudy of the PATH-BP trial (MacIntyre *et al.*, 2022). It was a single-center, randomized, double-blind, placebo-controlled, investigator-initiated crossover study. The aim of the study was to assess the impact of regular paracetamol treatment on blood pressure (BP) in individuals with hypertension during a 2-week period. The study was performed in the University of Edinburgh’s Clinical Research Centre (Western General Hospital, Edinburgh, UK). The study protocol was approved by the East of Scotland Research Ethics Service (13/ES/0087) and the Medicines and Healthcare products Regulatory Agency (2013-003204-40). It was registered with the U.S. National Institutes of Health (https://clinicaltrials.gov; Unique identifier: NCT01997112) and European Union Drug Regulating Authorities Clinical Trials Database (https://www.clinical-trialsregister.eu; Unique identifier: 2013-003204-40).

##### Study population

To be eligible for the study, individuals had to be aged ≥18 years of age and hypertensive. Individuals had to be either treated for hypertension, with an average daytime ambulatory BP of <150/95 mm Hg on stable doses of ≥1 antihypertensive medication, or untreated with an average daytime ambulatory BP ≥135/85 mm Hg and <150/95 mm Hg. Individuals with a history of ischemic heart disease, heart failure, cerebrovascular disease, liver impairment (ALT >50 IU/l), chronic kidney disease staged III–V, or suicidal ideation were excluded from the study. Individuals were also excluded if they weighed <55 kg or were regularly taking acetaminophen/paracetamol, nonsteroidal anti-inflammatory drugs (NSAIDs), corticosteroids, or oral anticoagulants. Written informed consent was provided by all participants prior to study involvement.

Screening was conducted for 204 local participants, and 110 participants were randomized onto the PATH-BP study (MacIntyre *et al.*, 2022). The dropout rate was less than 10%, with 7 participants not completing both arms of the study. Consequently, 103 participants were included in the modified intention-to-treat analysis, ensuring a balanced representation across all baseline characteristics in the study group.

The inclusion criterion for this biomarker substudy was a greater than 50% increase in ALT activity from baseline on paracetamol treatment. This was the definition of an ALT increase used in the SNAP Trial of novel paracetamol treatment regimens to identify potential liver injury in patients taking paracetamol in overdose (Bateman *et al.*, 2014).

##### Study protocol

Participants were randomly assigned to receive either 1 g paracetamol 4× daily (the maximum recommended daily dose and a commonly prescribed dose for chronic pain in the UK) or matched placebo for 2 weeks. Following a 2-week washout period, participants crossed over to the other treatment arm for an additional 2 weeks of treatment. The treatment order was randomized, with concealed allocation, using a random block design, and participants were assigned to receive drug then placebo, or placebo then drug. Blood samples were taken at 4 visits during each arm of the study (days 0 [pretreatment] 4, 7, and 14). All researchers and participants were blinded to treatment throughout the study.

##### Study outcomes

Study outcomes included standard clinical biochemistry parameters. ALT was measured in the NHS Lothian Biochemistry Laboratory. Total K18 (necrosis and apoptosis related forms) was measured using the Peviva M65 classic ELISA (Bioaxxes, Tewkesbury, UK). miR-122 was measured by PCR. GLDH was measured by its oxoglutarate reduction activity. Details of the biomarker measurement is described in the Supplementary Methods.

##### Statistical analysis

Data were statistically analyzed with GraphPad Prism 10 software (La Jolla, California). The fifth and 95th quartiles were derived to calculate reference intervals for each biomarker. Statistical significance for the performance of each biomarker was determined by Wilcoxon test. Pearson correlation was reported to evaluate the biomarkers in the context of ALT levels.

## Results

In the PATH-BP trial, a total of 204 participants were screened and 103 subjects were randomized onto the study between September 2014 and June 2019 (MacIntyre *et al.*, 2022). Supplementary Figure 1 presents the change in ALT activity in 103 randomized trial participants on placebo and paracetamol treatment. Of these 103 patients, 49 patients reached the predefined inclusion criteria of a greater than 50% increase in ALT activity from baseline on paracetamol treatment. The demographics of these 49 patients are presented in Table 1, alongside the 103 patients that were randomized into the PATH-BP trial. There were no substantial differences between the entire trial population and the substudy. In the 49 patients who reached the inclusion criteria, the median peak fold increase from baseline in ALT was 2.2 (IQR1.8–2.9, range 1.5–6.9). In the placebo arm, only 8 of the 49 patients had a 50% increase in ALT. Baseline values were comparable for all biomarkers when participants received paracetamol first (paracetamol then placebo baseline: ALT 21.3 ± 7.2U/l V 23.6 ± 10.4U/l, K18 11.4 ± 5.5 ng/ml V 11.6 ± 5.7 ng/ml, miR-122 4.6 ± 3.2fM V 4.9 ± 2.6fM and GLDH 8.7 ± 4.8U/l V 9.7 ± 2.9U/l) and when placebo was received first (placebo then paracetamol baseline: ALT 20.9 ± 7.6U/l V 18.6 ± 4.3U/l, K18 10.4 ± 4.4 ng/ml V 10.7 ± 4.3 ng/ml, miR-122 3.8 ± 2.4fM V 3.2 ± 2.1fMm, and GLDH 7.3 ± 2.4U/l V 8.0 ± 2.4U/l).

**Table 1.** Patient demographics for the PATH-BP Trial sub-population with ALT activity increase in paracetamol arm 50% or higher from baseline, and the full PATH-BP population.

		Sub-study—placebo then paracetamol		Substudy—paracetamol then placebo		PATH-BP—paracetamol then placebo		PATH-BP—placebo then paracetamol	
		*N*	%	*N*	%	*N*	%	*N*	%
Total		25		24		53		50	
Age years: mean (SD)		62.7 (7.6)		62.0 (9.0)		61.1 (7.9)		62.1 (8.1)	
Sex	Male	19	76	16	67	38	72	41	82
Smoking status	Current	0	0	1	4	2	7	1	2
	Ex-smoker	10	40	4	17	17	32	20	40
	Never	15	60	19	79	34	64	29	58
Daytime SBP/DBP (mmHg) (±SD)		136.4 (10.3)	83.2 (9.7)	131.5 (9.2)	81.8 (6.5)	132.8 (10.5)	81.2 (8.0)	133.9 (10.3)	81.7 (7.9)
24-h SBP/DBP (mmHg) (±SD)		130.0 (9.6)	79.0 (9.0)	128.5 (8.3)	77.4 (5.8)	126.5 (9.8)	76.8 (7.5)	127.4 (9.6)	77.3 (7.0)
On treatment for hypertension		17	68	15	63	38	72	31	62
Antihypertensive treatment—no. (%)									
	ACE inhibitor	7	28	9	38	19	36	12	20
	Angiotensin receptor blocker	7	28	5	21	18	34	15	30
	Calcium channel blocker	6	24	3	13	9	17	13	26
	Diuretic	9	36	5	21	12	23	16	32
	Beta-blockers	1	4	1	4	4	8	3	6
Number of antihypertensives—no. (%)									
	No antihypertensives	10	40	7	29	15	28	19	38
	1 drug	9	36	6	25	20	38	10	20
	2 drugs	4	16	9	38	12	23	14	28
	3 drugs	2	8	2	8	6	11	7	14

**Figure 1.**
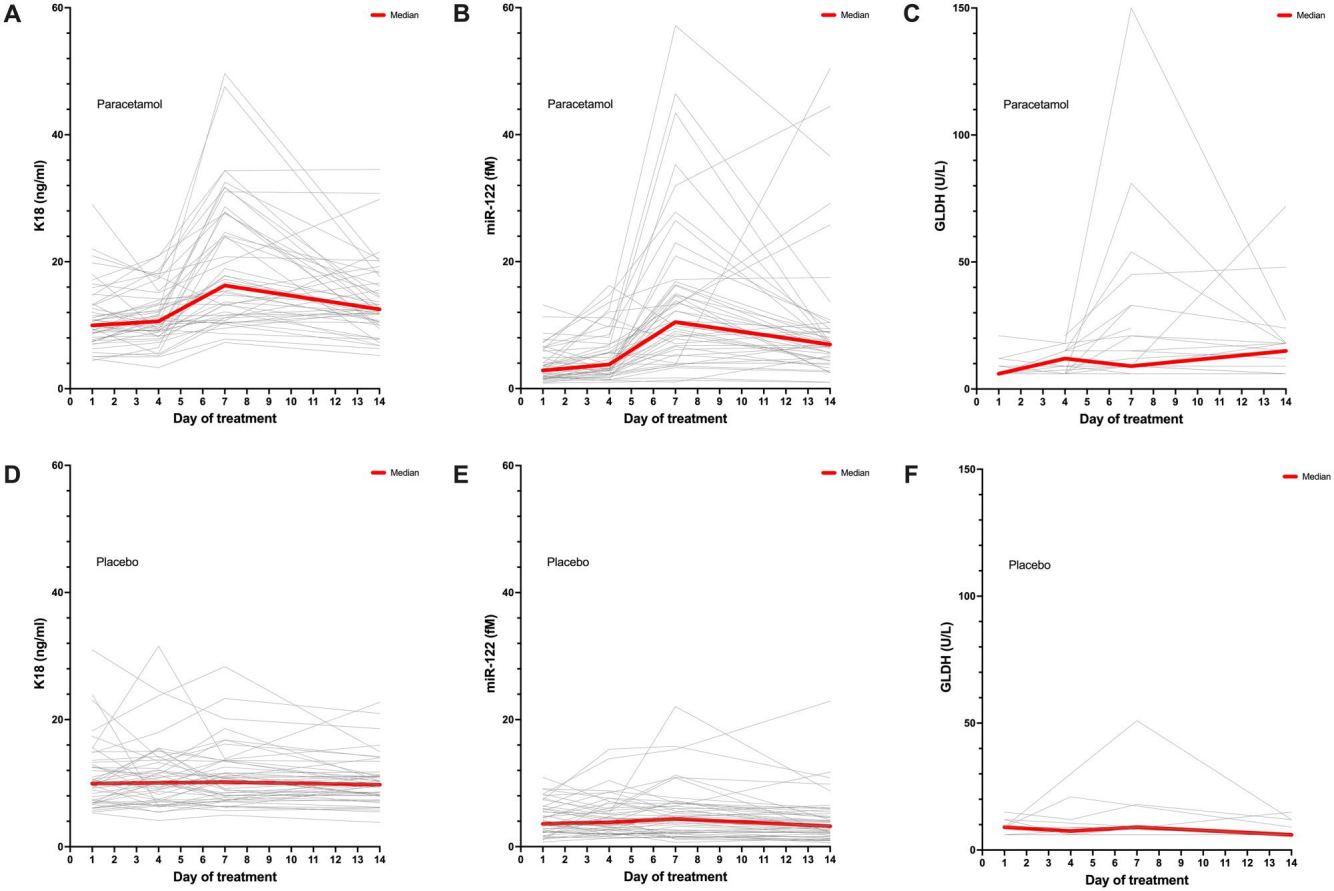
Change in serum biomarkers over 14 days of treatment with paracetamol (1 g 4 times per day) (A–C) or matched placebo (double-blind randomized trial) (D–F). Each line is a patient. The solid line is the population median. Population presented are patients from PATH-BP Trial with a 50% or more increase in peak ALT from baseline on paracetamol treatment. The concentration of GLDH was below the lower limit of detection (<5 U/l) in 261 out of 392 samples and these samples were excluded from the analysis.

**Figure 2.**
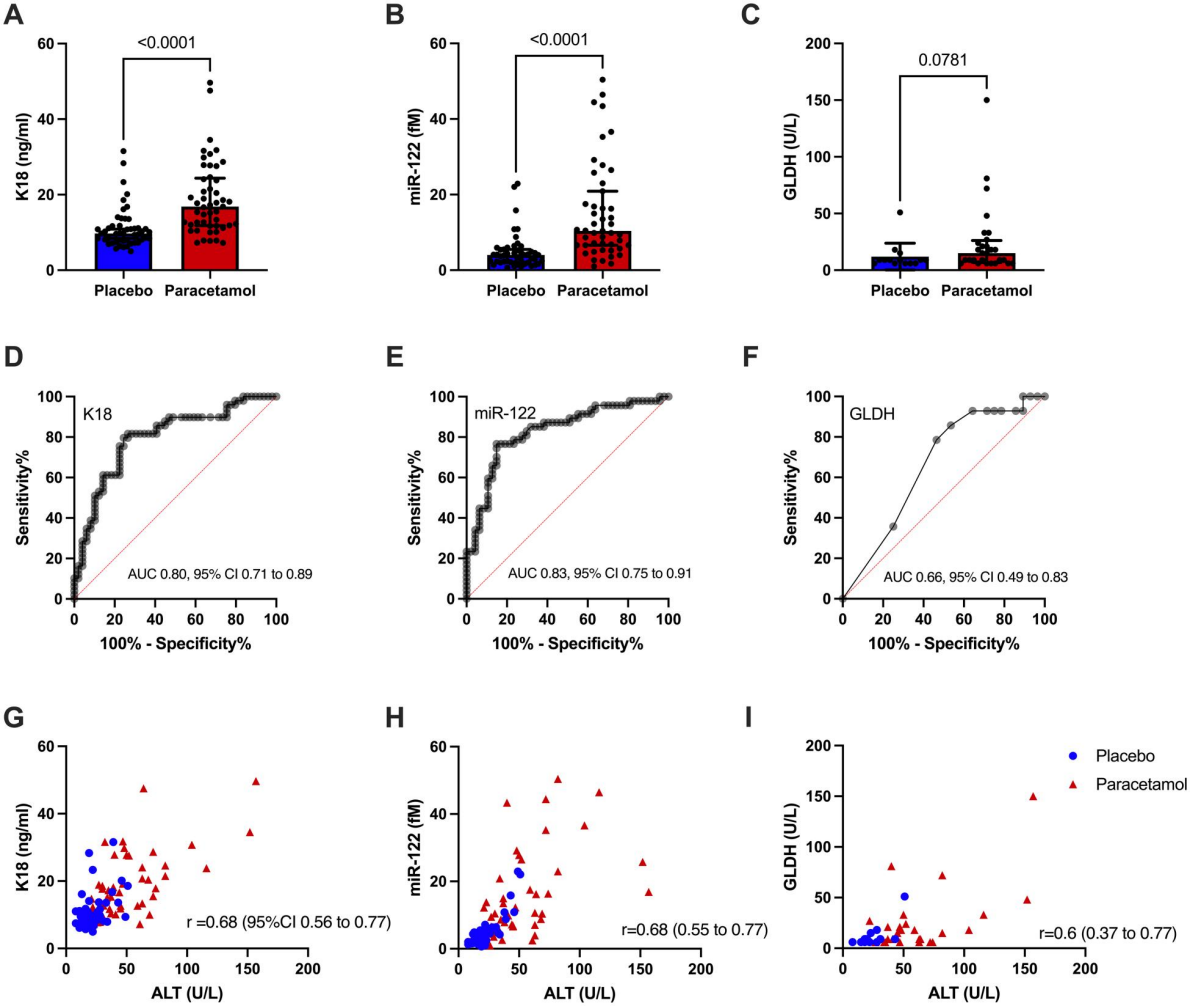
Biomarker performance at the trial visit at time of peak ALT in paracetamol treatment arm. A–C, biomarker concentration in placebo and paracetamol treatment arms. Significance determined by Wilcoxon test. Each dot is a participant. Bars show median and IQR. D–F, ROC curve analysis for biomarkers. AUC = area under curve. G–H, Biomarker correlation with ALT. Dots are placebo treatment, triangles are paracetamol treatment. Pearson *r* values are reported. The concentration of GLDH was below the lower limit of detection (<5 U/l) in 261 out of 392 samples and these samples were excluded from the analysis.

The 3 novel biomarkers (K18, miR-122, and GLDH)—that were previously identified as being the optimal panel for DILI identification after acute paracetamol overdose (Llewellyn *et al.*, 2021) were measured in the paracetamol and placebo arms of the 49 patients with an ALT increase with chronic paracetamol dosing (Figure 1). The concentration of GLDH was below the lower limit of detection (<5 U/l) in 261 out of 392 samples and these samples were excluded from the analysis. All 3 novel biomarkers were increased on the day of peak ALT (K18 paracetamol arm: 18.9 ± 9.7 ng/ml V placebo arm: 11.1 ± 5.4 ng/ml, ROC-AUC = 0.80, 95% CI 0.71–0.89, *p* < .0001, miR-122 15.1 ± 12.9fM V 4.9 ± 4.7fM, ROC-AUC = 0.83, 95% CI 0.75–0.91, *p* < .0001, and GLDH 24.6 ± 31.1U/l V 12.0 ± 11.8U/l, ROC-AUC = 0.66, 95% CI 0.49–0.83, *p* = .09) (Figure 2). All 3 biomarkers had a significant correlation with ALT activity: K18 Pearson *r* = 0.68 (95% CI 0.56–0.77); miR-122 *r* = 0.68 (0.55–0.77); GLDH *r* = 0.6 (0.37–0.77) (Figure 2).

**Figure 3.**
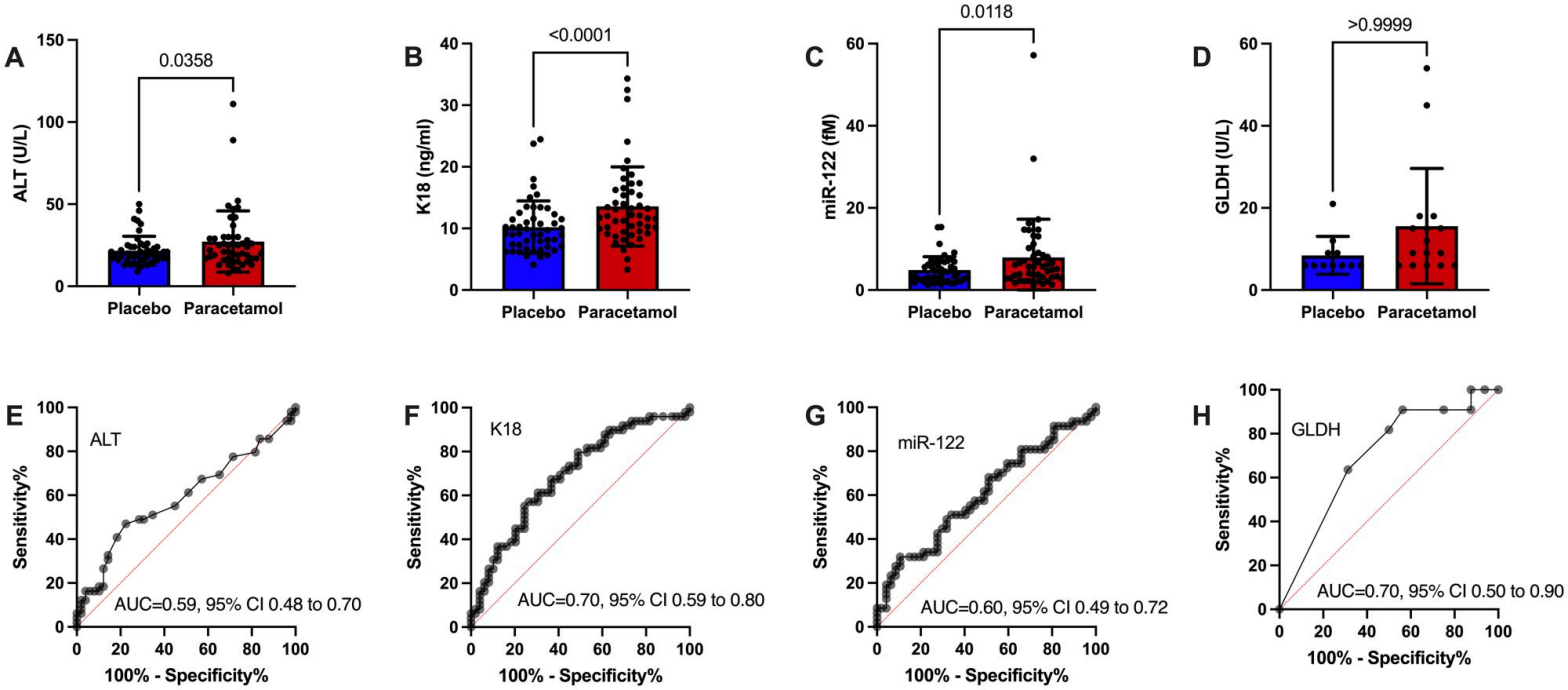
Biomarker performance at visit preceding the visit with measurement of peak ALT in paracetamol treatment arm. A–D, Biomarker concentration in placebo and paracetamol treatment arms. Significance determined by Wilcoxon test. Each dot is a participant. Bars show median and IQR. E–H, ROC curve analysis for biomarkers. AUC = area under curve. The concentration of GLDH was below the lower limit of detection (<5 U/L) in 261 out of 392 samples and these samples were excluded from the analysis.

In the setting of acute single paracetamol overdose and staggered overdose, multiple studies have demonstrated that K18 and miR-122 can identify patients who will develop liver injury before ALT has increased (Dear *et al.*, 2018). In this study, we had a population of patients with an ALT increase following chronic therapeutic dosing of paracetamol with a placebo arm to control for fluctuations in biomarker concentration which are unrelated to the study drug. Next, we compared the performance of the 3 novel biomarkers with ALT with regard to distinguishing paracetamol from placebo treatment at the visit preceding the peak ALT increase (mean 3 days prior to peak ALT). ROC curve analysis demonstrated that K18 had the highest sensitivity and specificity with regard to predicting an increase in ALT (K18 ROC-AUC = 0.70, 95% CI 0.59–0.80, *p* = .001; miR-122 ROC-AUC = 0.60, 95% CI 0.49–0.72, *p* = .08, ALT ROC-AUC = 0.59, 95% CI 0.48–0.70, *p* = .12; GLDH ROC-AUC = 0.70, 95% CI 0.50–0.90, *p* = .08) (Figure 3).

Published studies have reported higher variability in miR-122 than other DILI biomarkers (Church *et al.*, 2019). Therefore, we assessed the inter and intravariability of ALT and the 3 novel biomarkers in the placebo arm of the trial (Table 2). Intrasubject variability was determined using the placebo arm data across the 4 time points—ALT had the lowest variability (12.9% CV). Intersubject variability was similar for ALT and K18 (42.9%). miR-122 variability was higher both intrasubject (30.6%) and intersubject (74.1%). This is lower than reported by Church *et al.* (2019) (90.9% intersubject CV and 93.6% intrasubject CV). Variability in GLDH was not calculated given the high number of samples with unmeasurably low activity.

**Table 2.** Biomarker characteristics in the placebo treatment arm (*n* = 49).

Biomarker	Median	IQR	5% Percentile (LLN)	95% Percentile (ULN)	Intrasubject variation (CV %)	Intersubject variation (CV %)
ALT (IU/l)	19.0	16.0–24.8	11.9	43.3	12.9	42.9
K18 (ng/ml)	10.0	7.5–12.5	5.7	21.2	18.0	43.0
miR-122 (fM)	3.8	2.2–6.0	1.2	10.9	30.6	74.1

GLDH not included due to number of measurements below LLoQ.

## Discussion

This article presents the results of a biomarker analysis conducted on samples from a placebo-controlled, double-blind, crossover trial of paracetamol therapeutic dosing (MacIntyre *et al.*, 2022). This served as a model for an early-phase, multiple-dosing clinical trial with an investigational medicinal product that has a potential for causing DILI. The data reveal that there was an increase in ALT activity in patients receiving paracetamol compared with their treatment with placebo. The study evaluated 3 novel biomarkers selected based on their performance in the context of paracetamol overdose and found that K18 has most potential for predictive purposes and exhibited relatively low variability.

This study was conducted as a substudy of the PATH-BP trial, in which 110 individuals were randomized to receive 1 g paracetamol 4 times daily or a matched placebo for 2 weeks, followed by a 2-week washout period before crossing over to the alternate treatment. The primary outcome measure was blood pressure. A predefined cut-off of greater than 50% increase in ALT activity from baseline was used to select patients for this substudy. This cut-off was chosen based on its prior use in the SNAP Trial as a measure of potential liver injury (Bateman *et al.*, 2014). Of the 110 patients, 49 met the inclusion criteria for increased ALT activity. Although this cut-off increase was modest, 29 patients experienced a doubling of ALT activity from baseline on paracetamol, and 6 patients had a 4-fold increase in ALT. Similar to the PATH-BP trial, previous clinical studies have reported an increase in ALT activity following therapeutic dosing (Heard *et al.*, 2014; Watkins *et al.*, 2006). The significance of an increase in ALT activity during therapeutic paracetamol dosing is unclear; many of the patients with an ALT increase at day 7 have an ALT activity nearer baseline by day 14 (Supplementary Figure 1). Paracetamol has a dose-dependent intrinsic risk for DILI, and these ALT increases could be a warning of increased risk with dose escalation. However, clinically significant liver injury on paracetamol at therapeutic dosing is very rare, likely affecting only about 1 in 3 million patients treated for a year (Gulmez *et al.*, 2015).

The increase in ALT observed in this study offered an opportunity to assess the efficacy of novel biomarkers in a multiple dosing study. Among the biomarkers evaluated was K18, which is a mechanistic biomarker of liver injury and provides information on the type of cell death. In apoptosis, the caspase-cleaved form of K18 (cc-K18) is released early during cellular structural rearrangement, whereas in necrosis, the full-length form of K18 (FL-K18) is passively released upon cell death. As paracetamol-induced liver injury is predominantly necrotic with a component of apoptosis, total K18 was evaluated to identify liver injury. Another biomarker evaluated was miR-122, a 22-nucleotide microRNA that is highly specific to the liver and is released from necrotic hepatocytes, resulting in elevated concentrations in the bloodstream (Starkey Lewis *et al.*, 2011). Although miR-122 is an early marker of liver-specific damage, its use may be limited due to higher inter- and intraindividual variability and a potentially short half-life (Church *et al.*, 2019). Glutamate dehydrogenase (GLDH) was evaluated as an early marker of liver-specific mitochondrial damage (McGill *et al.*, 2012). The results showed that all 3 novel biomarkers increased when measured at the visit with peak ALT on paracetamol compared with the same visit on placebo treatment and correlated with ALT. This is consistent with published data on paracetamol and non-paracetamol DILI.

Serum samples collected prospectively in PATH-BP allowed testing whether any of the 3 markers were increased (compared with placebo) on the visit preceding the peak ALT increase, which was, on average, 3 days prior. Only K18 showed significant predictive value when analyzed using ROC curves. This suggests that K18 may be useful in the early detection of DILI risk in patients prescribed medications known to be potentially hepatotoxic. This finding is consistent with data from (1) patients who have taken a paracetamol overdose, which shows that K18 is a sensitive and specific predictor of subsequent liver injury despite treatment, and (2) from a trial of hospitalized volunteers given paracetamol for 7 days (Thulin *et al.*, 2014). This latter study did not have a placebo arm unlike PATH-BP, which provides evidence that the change in ALT is paracetamol-induced and not related to other trial related factors such as dietary changes.

In the setting of paracetamol overdose, miR-122 is a sensitive early biomarker of liver injury. The reduction in performance compared with K18 in this study may reflect the small peak ALT values (and less cell injury) and/or the higher variability of miR-122. However, the variability we report in this article is lower than previous reports and may not be significant when more hepatocellular injury is present (such as paracetamol hepatotoxicity). GLDH has been repeatedly demonstrated to have lower sensitivity compared with both K18 and miR-122 in acute overdose studies, consistent with the current study in repeated dosing.

There are limitations to this study. For this study, the increases in ALT were modest, however, it is probable that K18 would also predict more significant liver injury. This has been demonstrated in repeated studies following acute paracetamol overdose (Dear *et al.*, 2018). We did not measure marker concentrations in those patients without an increase in ALT on paracetamol treatment. However, the markers are well-established to distinguish patients with and without DILI in both paracetamol overdose and idiosyncratic injury. The assays used in this study are research laboratory based and not optimized for clinical practice (eg, the time to result is too long for acute decision-making), though point-of-care assays are in development. However, whether biomarker performance is maintained on different platforms remains to be confirmed. GLDH was below the limit of assay detection in 261 out of 392 samples. This limited conclusions about GLDH performance and higher sensitivity assays may improve biomarker performance in the context of early DILI diagnosis.

In summary, K18 was more sensitive than ALT, miR-122, and GLDH and has potential for the early identification of potential DILI in clinical trials.

## Supplementary Material

kfae031_Supplementary_Data
