# Supplementary material for: Cytokeratin-18 is a sensitive biomarker of alanine transaminase increase in a placebo-controlled, randomized, crossover trial of therapeutic paracetamol dosing (PATH-BP biomarker substudy)
Source: Toxicol Sci. 2024 Mar 23;199(2):203–9. doi: 10.1093/toxsci/kfae031 (PMC11131027; doi:10.1093/toxsci/kfae031)
Supplement: kfae031_Supplementary_Data [file kfae031_supplementary_data.zip › kfae031_Supplementary_Data/toxsci-23-0326-File005.docx]

**Cytokeratin-18 is a sensitive biomarker of alanine transaminase increase in a placebo-controlled, randomised, crossover trial of therapeutic paracetamol dosing (PATH-BP Biomarker sub-study)**

Kathleen M Scullion, Iain Macintyre, Sian Sloan-Dennison, Benjamin Clark, Paul Fineran, Joanne Mair, David Creasey, Cicely Rathmell, Karen Faulds, Duncan Graham, David J Webb, James W Dear

**Supplementary Methods**

**K18/M65**
The M65 ELISA was conducted at room temperature (24 ± 3°C). Reagents were prepared following manufacturer’s guidelines. Standards, high and low controls, and samples with unknown concentrations (25μl each) were added to the microplate in duplicate. To each well, 75μl of diluted M65 HRP Conjugate solution was added sequentially within 20 minutes. The microplate was sealed to prevent evaporation and contamination, then incubated on a shaker for 2 hours at 600 rpm to facilitate the binding of the M65 HRP Conjugate to the target analyte. Following incubation, the plate was washed five times with 400μl of wash solution per well using a plate washer. Subsequently, 200μl of TMB Substrate was added to each well. The plate was incubated in the dark at room temperature for 20 ± 1 minutes, allowing the enzymatic reaction to take place. To stop the reaction, 50μl of Stop Solution was added to each well, and the microplate was gently shaken for 10 seconds to ensure thorough mixing. Absorbance was measured after 5 minutes at 450nm. The recorded absorbance values served as indicators of the analyte concentrations in the samples.

To ensure quality control, each ELISA run underwent specific test procedures. Firstly, the provided standards were assessed to ensure a simple linear regression with an appropriate R-squared value, confirming the reliability of the standard curve. Secondly, all samples were expected to yield values that could be interpolated from the standard curve. In case this criterion was not met, samples were re-measured at a 1:1 dilution with Standard A to ensure accurate quantification of the analyte.

**miR-122**

*RNA Extraction*

RNA Extraction was carried out on human serum samples using a commercial kit (miRNeasy Serum/Plasma kit, Qiagen, Venlo, Netherlands) following manufacturer’s guidelines. Serum samples (50µl) were diluted with RNase free water (150µl). QIAzol Lysis Reagent (1ml) was then added to the diluted sample before mixing. The homogenate was incubated for 5 minutes at room temperature. Following this, 200µl chloroform was added and the sample was shaken vigorously for 15 seconds before incubating at room temperature for 3 minutes. The samples were then centrifuged for 15 minutes at 12,000 x *g* at 4°C. Next, the upper aqueous phase was transferred to a new collection tube, ensuring that any interphase was avoided. To this, 900μl of 100% ethanol was added and mixed thoroughly. The samples were then transferred into a RNeasy MinElute spin column and centrifuged at ≥8000 x *g* for 15 seconds at room temperature. Flow-through was discarded. Buffer RWT (700μl) was then added to the spin column and centrifuged, with the flow-through discarded. This was repeated with Buffer RPE and 80% ethanol (500μl). The spin column was centrifuged at full speed for 5 min to dry the membrane. To the dried column, 14μl RNase-free water was added directly to the centre of the membrane and centrifuged for 1 minute at full speed to elute the RNA.

*Reverse Transcription*

The microRNA-containing eluate was reverse-transcribed using the TaqMan microRNA Reverse Transcription kit (thermos Fischer Scientific, Waltham, Massachusetts, United States) following manufacturer’s instructions. The prepared RT Reaction Mix (3.5µL) and 5 µL of total RNA were combined in each well of a 96 well reaction plate and mixed thoroughly before being centrifuged briefly to collect the contents at the bottom of the wells. To this, 1.5 µL of 5x RT Primer was added to each well and the plate was sealed and centrifuged briefly. The reaction was performed using a thermal cycler following the manufacturer’s cycling conditions.

*PCR Amplification*

To prepare the PCR amplification, 9.4µL of the PCR Reaction Mix was added to each well of a 384 well plate followed by 0.6µL cDNA template, or nuclease–free water for non-template controls. The plate was sealed and centrifuged briefly to bring the PCR Reaction Mix to the bottom of the wells. Amplification was performed using a Roche Lightcycler, following the manufacturer’s cycling conditions.

**GLDH**

Frozen human serum samples were transported on ice to the Specialist Assay Service at Edinburgh University BioQuarter for analysis. GLDH concentration was determined using a commercial kit (DiaLab via Alpha Laboratories Ltd., Eastleigh, UK) following the manufacturers guidelines adapted for use on either a Cobas Fara or Cobas Mira analyser (Roche Diagnostics Ltd, Welwyn Garden City, UK).
