# Supplementary figures and images for: Cytokeratin-18 is a sensitive biomarker of alanine transaminase increase in a placebo-controlled, randomized, crossover trial of therapeutic paracetamol dosing (PATH-BP biomarker substudy)
Source: Toxicol Sci. 2024 Mar 23;199(2):203–9. doi: 10.1093/toxsci/kfae031 (PMC11131027; doi:10.1093/toxsci/kfae031)

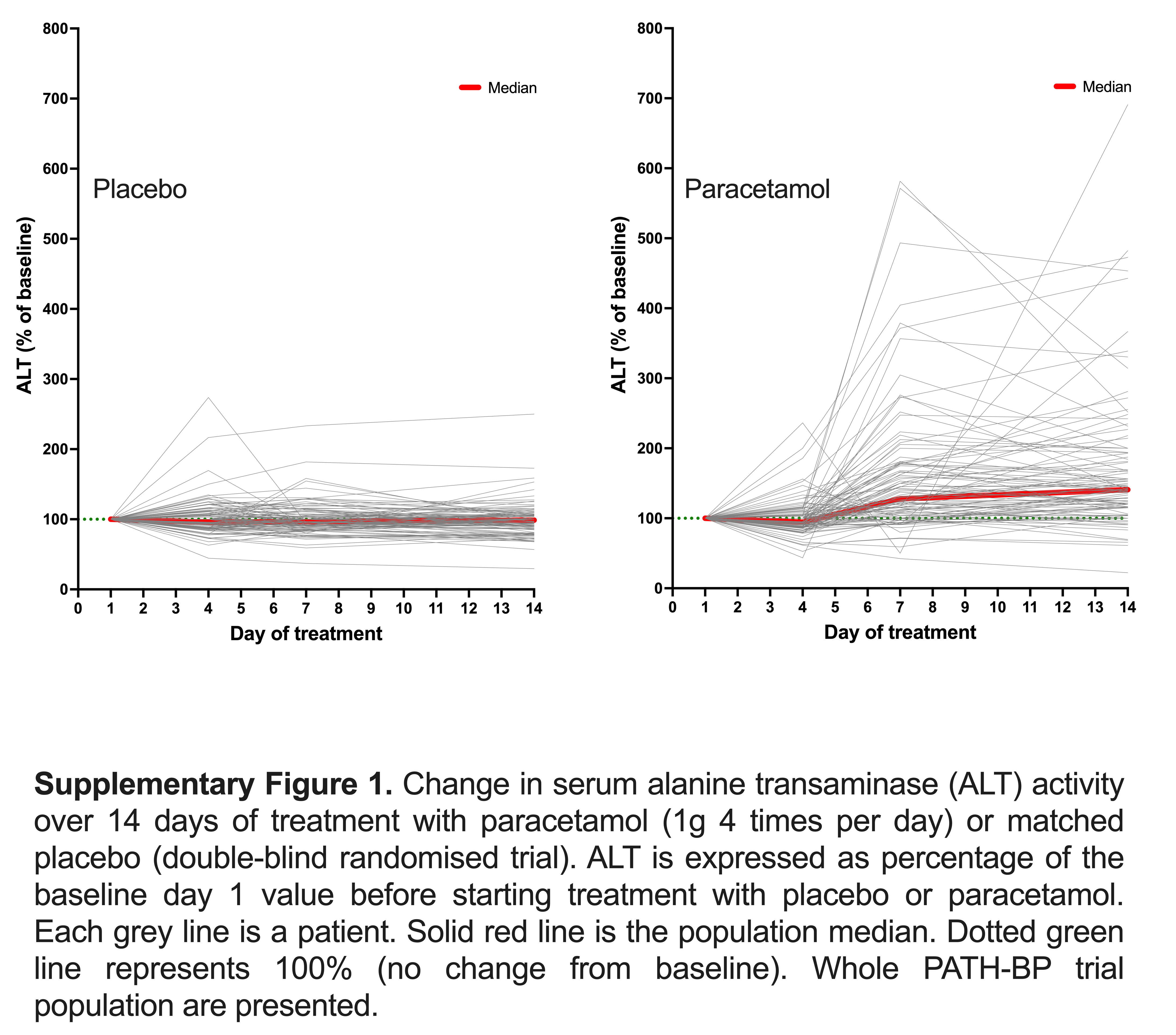

Supplement: kfae031_Supplementary_Data [file kfae031_supplementary_data.zip › kfae031_Supplementary_Data/toxsci-23-0326-File006.tiff]
